# Ion buffering and interface charge enable high performance electronics with organic electrochemical transistors

**DOI:** 10.1038/s41467-019-11073-4

**Published:** 2019-07-10

**Authors:** Paolo Romele, Matteo Ghittorelli, Zsolt Miklós Kovács-Vajna, Fabrizio Torricelli

**Affiliations:** Department of Information Engineering, University of Brescia, 25123 Brescia, Italy

**Keywords:** Electronic devices, Molecular electronics

## Abstract

Organic electrochemical transistors rely on ionic-electronic volumetric interaction to provide a seamless interface between biology and electronics with outstanding signal amplification. Despite their huge potential, further progress is limited owing to the lack of understanding of the device fundamentals. Here, we investigate organic electrochemical transistors in a wide range of experimental conditions by combining electrical analyses and device modeling. We show that the measurements can be quantitatively explained by nanoscale ionic-electronic charge interaction, giving rise to ion buffering and interface charge compensation. The investigation systematically explains and unifies a wide range of experiments, providing the rationale for the development of high-performance electronics. Unipolar inverters — universal building blocks for electronics — with gain larger than 100 are demonstrated. This is the highest gain ever reported, enabling the design of devices and circuits with enhanced performance and opening opportunities for the next-generation integrated bioelectronics and neuromorphic computing.

## Abstract (web summary)

The rationale design of optimized organic electrochemical transistors (OECTs) for next-generation bioelectronics requires further exploration of the underlying device physics. Here, the authors report the role of ionic-electronic charge interactions on OECTs and high-performance unipolar inverters.

## Introduction

Organic electrochemical transistors (OECTs) are iontronic devices where ions penetrate the semiconductor and dramatically modulate the electrical properties of the transistor channel. Owing to this bulk ionic-electronic interaction, OECTs provide a seamless interface between biology and electronics combining the benefits typical of organic material technologies—such as large-area deposition with simple and low-cost techniques, chemically-tunable properties, mechanical flexibility, softness, and biological compatibility^1–5^—with high signal amplification, ultra-low voltage operation, and stability in aqueous environment^6–8^. Fueled by this unique benefits combination, OECTs are gaining significant interest in numerous bioelectronic applications, including neural interfacing, electrophysiology, cell monitoring, enhanced ionic and biological sensing, neuromorphic devices, and neuron stimulation^9–17^. Despite the huge potential, the lack of understanding of the fundamental processes governing the device operation hinder further progress in the rational design of engineered and optimized devices for new and improved applications.

The operation of OECTs has been described for the first time by Bernards and Malliaras^18^ in 2007. Their pioneering work depicted OECTs as the combination of an electronic circuit that accounts for electronic transport in the organic semiconductor, and an ionic circuit that accounts for ionic transport in the electrolyte. Importantly, the model captured the key characteristic of OECTs, i.e., the volumetric response due to the ion penetration into the transistor channel. This point has been recently investigated by several experimental works^19–22^, showing that ions uptake from an electrolyte into a polymeric film results in a purely volumetric capacitance^19^. The linear dependence of the capacitance on the volume of the polymeric channel and the zero offset led to the conclusion that the ionic charges are uniformly distributed in the polymer and no significant ion accumulation at the polymer/electrolyte interface takes place^23^. On one hand, studies^24^ based on the modeling of cyclic voltammograms of the prototypical conducting polymer poly(3,4-ethylenedioxythiophene) doped with the polyelectrolyte poly(styrene sulfonate) (PEDOT:PSS) suggest that the capacitance originates from an electrical double layer (EDL) at the interface between the PEDOT phase and the PSS phase. In this direction, Tybrandt and coworkers^25^ show a modified drift diffusion model for the description of PEDOT:PSS based OECTs. On the other hand, several reports show that in conductive polymers the characteristic shape of the supercapacitive voltammograms is due to pseudo-capacitive processes involving faradaic reactions^26–29^. These findings are further corroborated by specific studies on OECTs, where the dedoping process is commonly explained as a faradaic reaction^30–33^. The redox model is usually invoked to explain the ion concentration dependent OECT transfer characteristics^31^ but a limited range of ion concentrations has been assessed. In this complex scenario, a comprehensive experimental and theoretical analysis identifying the key physical effects and providing a consistent picture of the OECT operation is highly desirable.

Here we investigate the ionic-electronic interaction in OECTs accounting for a very wide range of ion concentrations, channel thicknesses, and polymer charge densities. OECTs are systematically analyzed by combining electrochemical impedance spectroscopy, current-voltage characteristics, and device modeling. First, the analysis considers the widely studied PEDOT:PSS OECTs, enabling us to compare our results with the state of the art. Then, to prove the generality of our results, we modify the polymer formulation by changing the amount of fixed charges in the polyelectrolyte by about two orders of magnitude. Finally, the analysis is extended to a widely used accumulation mode material, namely poly(3-hexylthiophene-2,5-diyl) (P3HT). We show that the measurements can be quantitatively explained by an electrostatic bulk uptake of ions that compensate both fixed and mobile electronic charges. The key OECT characteristics can be quantitatively explained by ion buffering and interface charge compensation. The analysis unifies a wide range of experiments, explaining the ion concentration-dependent threshold voltage and the ion concentration-independent volumetric capacitance in OECTs. On the basis of this understanding, we demonstrate OECT unipolar inverters, ubiquitous building blocks for electronics, with gain and noise margin up to 107 and 82% of the theoretical limit, respectively. These are the best performances ever reported for the fundamental figures of merit of inverters and, importantly, are achieved basing on a device-aware circuit design approach.

## Results

### Device structure and electrical characteristics

**Fig. 1:**
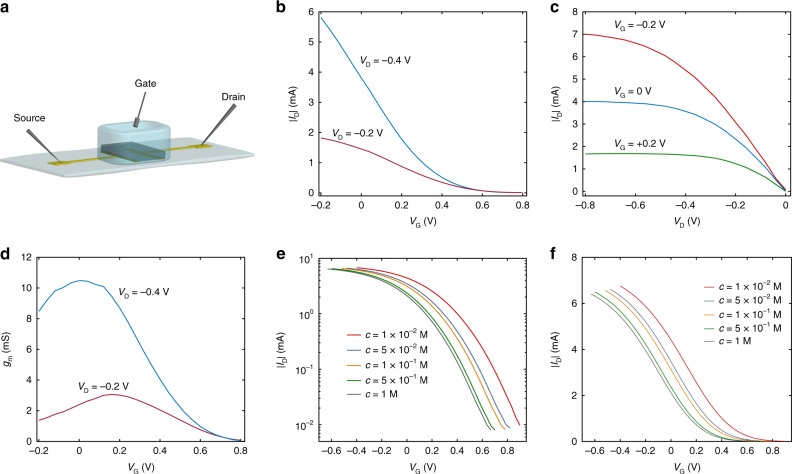
OECTs structure and electrical characteristics. **a** Schematic representation of an OECT. **b** Typical transfer characteristics in linear scale of an OECT. **c** Typical output characteristics of an OECT. The NaCl concentration is 100 mM. **d** Measured transconductance *g*_m_ = d*I*_D_/d*V*_*G*_, with a maximum *g*_m_ of about 10 mS at *V*_G_ = 0 V and *V*_D_ = −0.4 V. The transconductance normalized to the device geometry and drain voltage amounts to 60 S cm^−1^ V^−1^. **e**, **f** Typical transfer characteristics (semi-log scale and linear scale) measured at several NaCl concentrations and at *V*_D_ = −0.4 V. The device thickness is 2095 nm

A schematic representation of the OECT structure is depicted in Fig. 1a. A thin film of PEDOT:PSS is deposited by inkjet printing on a plastic polyethylene foil. Gold is used for source and drain electrodes. The transistor channel width and length are *W* = 1000 μm and *L* = 500 μm, respectively. The printing technique allows to easily vary the channel thickness (*t*) by printing multiple stacked polymer layers, ranging from 230 to 2300 nm. It is worth to note that the channel thickness is a key design parameter because it enables the tuning of the transistor capacitance and, in turn, of the transconductance^19^. As electrolyte, we used an aqueous solution of sodium-chloride (NaCl) at concentrations ranging from *c* = 1 10^−3^ M to *c* = 5 M. A PDMS well is used to spatially confine the electrolyte. The wide range of ion concentrations and polymer thicknesses here investigated is extremely relevant for OECT-based biological applications, sensing, and circuits^19,34–36^. The gate electrode is an Ag/AgCl pellet immersed into the electrolyte. Further details on the OECTs fabrication are provided in the Methods section.

Typical transfer and output characteristics of the fabricated devices are shown in Fig. 1b, c, respectively. By applying a positive gate voltage (*V*_G_) cations drift into the polymer, reduce the hole concentration and lower the drain current (*I*_D_). Analogously, when a negative *V*_G_ is applied previously injected cations drift out of the polymer while anions drift into the polymer, the hole concentration increases and this results in a larger *I*_D_. As shown in Fig. 1d the maximum transconductance normalized to the OECT geometries and drain voltage is larger than 60 S cm^−1^ V^−1^ at *V*_G_ = 0 V and *V*_D_ = −0.4 V, in agreement with state-of-the-art OECTs^6^. The extremely large transconductance, resulting from the ionic-electronic interaction through the bulk of the polymeric channel, is a hallmark of OECTs.

### Analysis of the main OECT parameters

To investigate the ionic-electronic interaction in OECTs, we measured the transfer characteristics (*I*_D_–*V*_G_) by varying the ion concentration *c*. Figure 1e, f shows that the *I*_D_–*V*_G_ characteristics systematically shift to more negative voltages with increasing ion concentrations. To gain insight on the physical mechanisms underlying the device operation, we reproduced the electrical characteristics of the OECTs with the drain current model proposed by Bernards and Malliaras^18^, that in the case of linear operation reads^18,37^:

1 $$I_{\mathrm{D}} = \Gamma \left[ {\left( {V_{\mathrm{T}} - V_{\mathrm{G}}} \right)V_{\mathrm{D}} + \frac{{V_{\mathrm{D}}^2}}{2}} \right]$$

where

2 $$\Gamma = \frac{{Wt}}{L}\mu C_{\mathrm{v}}$$

The drain current depends on the geometrical and physical device parameters, namely polymer width (*W*), length (*L*), thickness (*t*), hole mobility (*μ*), volumetric capacitance (*C*_v_), and threshold voltage (*V*_*T*_), which is defined as:

3 $$V_{\mathrm{T}} = V_{\mathrm{P}} - V_{{\mathrm{SH}}}$$

where *V*_P_ *=* *q p*_0_ *C*_v_^−1^ is the pinch-off voltage, *q* is the elementary charge, *p*_0_ is the intrinsic doping of the semiconductor, and *V*_SH_ accounts for the voltage shift as a function of the ion concentration *c*. It is worth noting that *V*_SH_ can be attributed to both the gate/electrolyte and electrolyte/semiconductor interfaces.

**Fig. 2:**
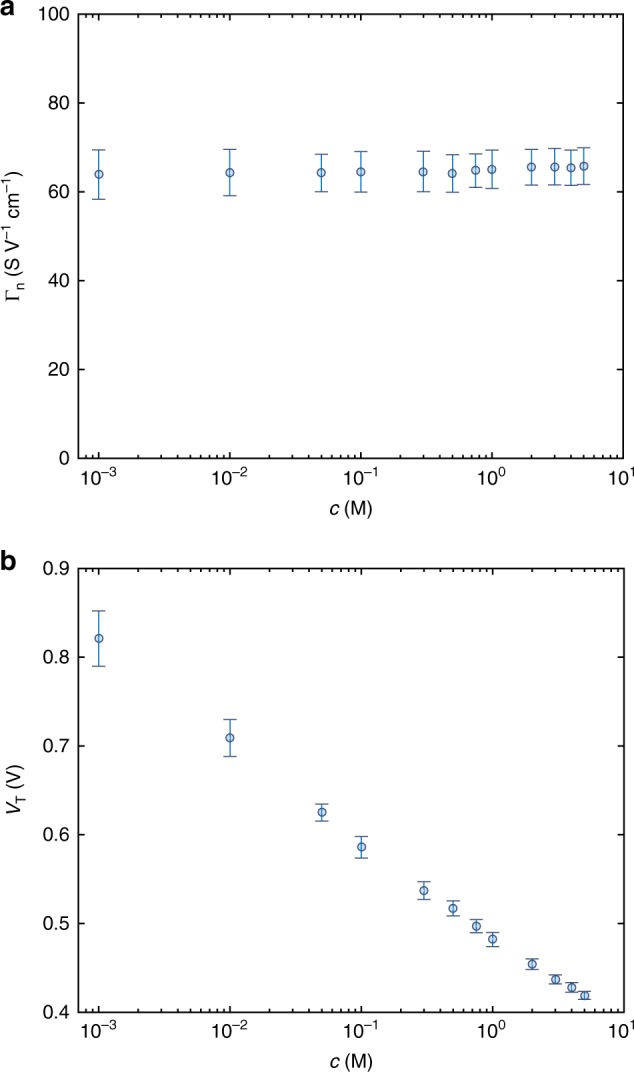
OECT parameters as a function of the ion concentration. **a** Normalized conductivity *Γ*_n_ = *Γ* *L* *W*^−1^ *t*^−1^ as a function of the ion concentration. **b** Threshold voltage *V*_T_ as a function of the ion concentration. Circles are the mean value and error bars are the standard deviation calculated by modeling ten OECTs with various thicknesses

According to Eq. (1), the drain current depends on the applied voltages (*V*_G_ and *V*_D_) and on the device parameters *Γ* and *V*_T_. *Γ* and *V*_T_ can be obtained by fitting the transfer characteristics in the linear regime. The modeling is systematically performed on OECTs with various channel thicknesses and the transfer characteristics are reproduced. Supplementary Figure 1 shows the modeling of the *I*_D_-*V*_G_ as a function of *c*. It is worth to note that we focus on a narrow range of the transfer characteristics since this enables a reliable extraction of the model parameters and ensures excellent stability of the OECT characteristics^38,39^ (Supplementary Fig. 2). The slope of the linear least square approximation of the *I*_D_*–V*_G_ provides *Γ*. Figure 2a shows *Γ* normalized to the channel geometries (viz. *Γ*_n_ = *Γ L W*^−1^ *t*^−1^) as a function of *c*. For each *c* the mean value and standard deviation of *Γ*_n_ are calculated by modeling OECTs with several thicknesses. We found that *Γ*_n_ is independent of *c* and, according to Eq. (2), it follows that *C*_v_ is independent of *c*. The analysis is further corroborated by reproducing the OECT transfer characteristics with the drain current model recently proposed by Friedlein and co-workers^40^ (Supplementary Note 1), which accounts for a non-uniform mobility in OECTs and provides superior fitting performance of the saturation region of operation (Supplementary Fig. 2a). Supplementary Figure 2d shows *Γ*_n_ extracted by fitting the OECT characteristics with the Friedlein model, confirming that *C*_v_ is independent of *c*.

A *c*-independent *C*_v_ is crucially important because in the case of an EDL a capacitance affected by the counterion concentration is expected^41,42^. To investigate this point, we perform electrochemical impedance spectroscopy (EIS) measurements by systematically varying the counterion concentration. The OECT impedance spectra as a function of *c* are shown in Supplementary Fig. 3. In order to obtain *C*_v_, for each *c* and *t* we modeled the measured impedance as a function of the frequency with the Randles equivalent circuit that, in the case of an OECT, is composed of a resistor *R*_s_ in series with the parallel of a resistor *R*_p_ and a capacitor *C* ^19^. In this model, *R*_s_ is the electrolyte resistance, *R*_p_ depends on the reactions at the working electrode, and *C* accounts for the ion accumulation at the working electrode^43^. The OECT volumetric capacitance is calculated as *C*_v_ *=* *C v*^−1^ where *v* *=* *W L t* is the total volume of the OECT channel. *C*_v_ and *R*_s_ as a function of *c* are shown in Supplementary Fig. 4. In all cases we found that *C*_v_ is independent of the ion concentration and results *C*_v_ = 44 ± 2 F cm^−3^. This value is in agreement with the state-of-the-art^19,25^.

**Fig. 3:**
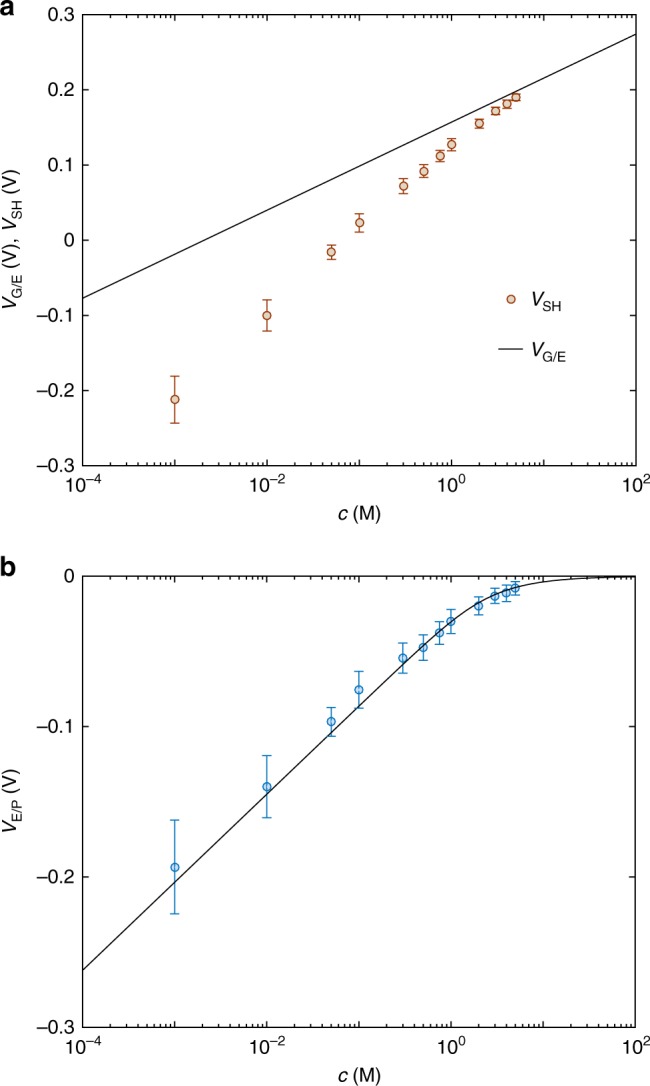
Potential at the interfaces as a function of the ion concentration. **a** Measured threshold voltage shift *V*_SH_ as a function of the ion concentration (symbols) and Nernst potential at the gate/electrolyte interface *V*_G/E_ (vs. SHE) calculated with Eq. (5) (solid line). **b** Potential at the electrolyte/polymer interface *V*_E/P_ as a function of the ion concentration. Symbols are the measurements and solid line is calculated with Eq. (6). Circles are the mean value and error bars are the standard deviation calculated by modeling ten OECTs with various thicknesses

To further investigate the origin of the ion-dependent drain current displayed in Fig. 1e, f, we analyzed *V*_T_ as a function of *c*. The mean value and standard deviation are extracted from OECTs with several thicknesses at each ion concentration. Figure 2b shows that *V*_T_ decreases with increasing ion concentration. According to the previous analysis, the pinch-off voltage is independent of the ion concentration (*V*_P_ = *q p*_0_ *C*_v_^−1^) provided that *p*_0_ is independent of *c*. To this aim, we monitored the polymer conductivity as a function *c* by measuring the drain current of an OECT when the gate electrode is not immersed into the electrolyte. We found that *I*_D_ is independent of *c* (Supplementary Fig. 5) and therefore the variation of *V*_T_ as a function of c can be ascribed to *V*_SH_. Figure 3a (symbols) shows the extracted *V*_SH_ as a function of c. *V*_SH_ accounts for the voltage drop at both the gate/electrolyte (*V*_G/E_) and the electrolyte/polymer (*V*_E/P_) interfaces, and reads:

4 $$V_{{\mathrm{SH}}} = V_{{\mathrm{G}}/{\mathrm{E}}} + V_{{\mathrm{E}}/{\mathrm{P}}}$$

Focusing on the gate-electrolyte interface, it is worth to note that we used an Ag/AgCl pellet as gate electrode, which is a non-polarizable quasi-reference electrode. According to the Nernst equation, the potential drop at the gate-electrolyte interface results^44^:

5 $$V_{{\mathrm{G}}/{\mathrm{E}}} = \frac{{k_{\mathrm{B}}T}}{q}\log c$$

where *k*_B_ is the Boltzmann constant and *T* is the temperature. Figure 3a (full line) shows the voltage drop at the gate/electrolyte interface calculated with Eq. (5). At large ion concentrations *V*_G/E_ (full line) is close to *V*_SH_ (symbols), while at small ion concentrations *V*_G/E_ is significantly larger than *V*_SH_. This indicates that the contribution of the electrolyte/polymer interface cannot be neglected and it can be calculated with Eq. (4), viz. *V*_E/P_ *=* *V*_SH_ − *V*_G/E_.

### The role of the bulk charge interaction

**Fig. 4:**
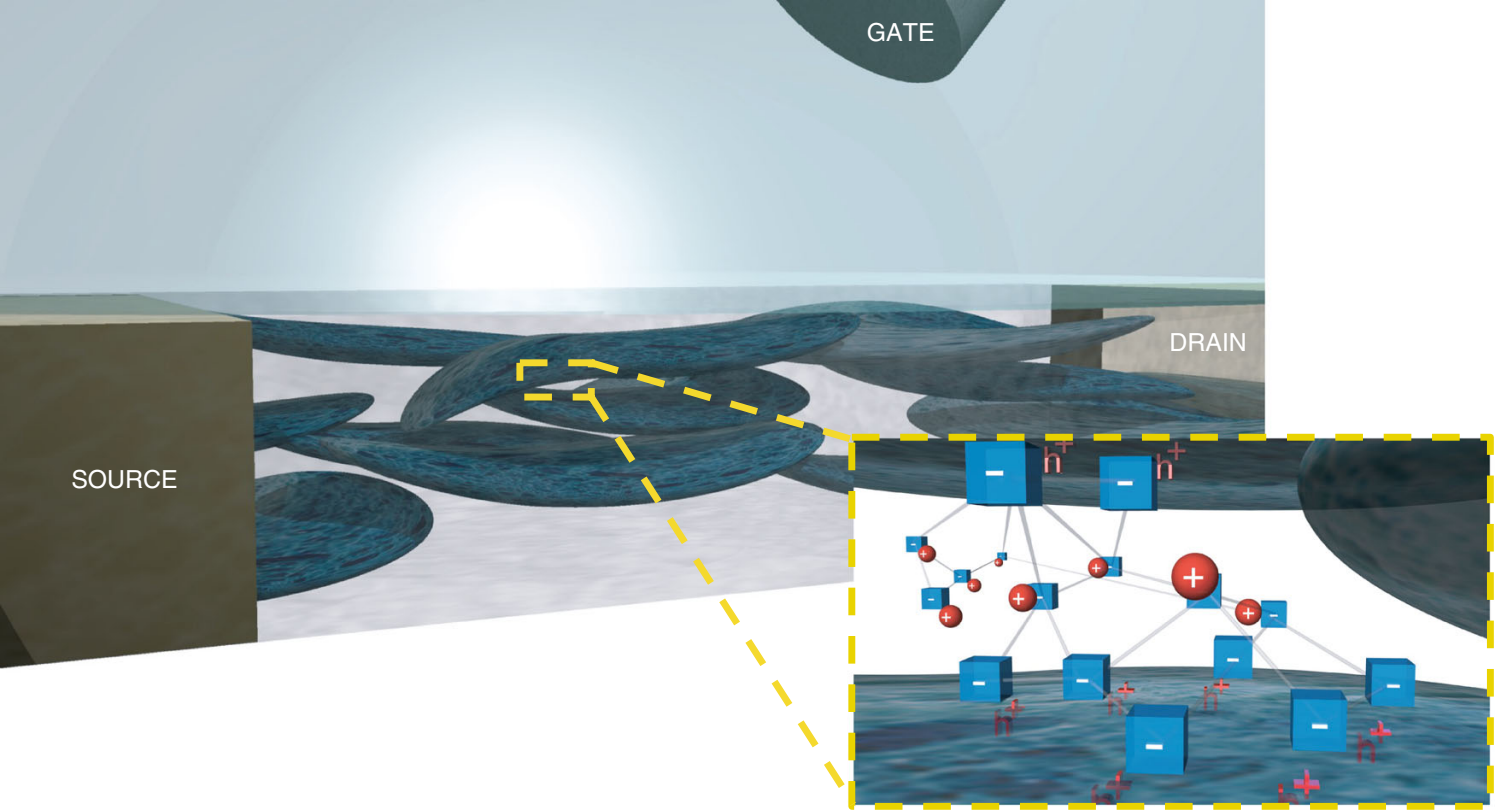
Schematic material-view of a PEDOT:PSS OECT. The polymeric channel is depicted by PEDOT-rich grains (dark blue) hosted by a PSS-rich matrix (gray). The zoom shows the ionic–electronic interaction in the PEDOT:PSS channel. Fixed anions in the PSS polyelectrolyte (blue cubes) are electrostatically compensated both by holes in the PEDOT semiconductor (red h^+^) at the PEDOT/PSS interface and by mobile cations (red spheres) in the bulk of the PSS

Figure 3b (symbols) shows the voltage drop at the electrolyte/polymer interface (*V*_E/P_) as a function of *c*. Supplementary Figure 6 shows that *V*_E/P_ extracted with the Friedlein et al. model fully agrees. *V*_E/P_ is large and negative (*V*_E/P_ ≈ −0.2 V) at the minimum ion concentration *c* = 10^−3^ M, it increases with a Nernstian slope of 59 mV dec^−1^ up to *c* = 5 10^−1^ M, and at larger concentrations *V*_E/P_ is close to zero. This can be explained as follows. In OECTs, ions dissolved in the electrolyte can flow into the bulk of the polymeric channel. In the case of PEDOT:PSS OECTs, the channel is made of a polymer blending composed of a PSS-rich matrix hosting nanometric sized PEDOT-rich grains^20,24,25^, as schematically depicted in Fig. 4. The electronic (hole) conduction takes place in the PEDOT (blue islands in Fig. 4) while ionic conduction is provided by the PSS polyelectrolyte (light-gray regions in Fig. 4). Indeed, when in contact with an electrolyte, the PSS matrix swells and allows the hydrated ions to penetrate the polymeric blend^20,45^. The polyelectrolyte/electrolyte interface acts as a semi-permeable membrane, which can be penetrated by the mobile ions provided by the electrolyte solution (red spheres in Fig. 4) but cannot be crossed by the SO_3_^−^ fixed anions of the PSS (blue cubes in Fig. 4). As a consequence, the fixed charges in the bulk of the polyelectrolyte (*N*_fix_) are electrostatically compensated by cations provided by the electrolyte. More in detail, at low electrolyte concentrations (*c* « *N*_fix_) cations drift into the polymer and compensate the fixed negative charges. At the equilibrium, the cation concentration in the polymer (*c*^PSS^_+_) is equal to *N*_fix_ independently of *c* and a negative potential rises at the polyelectrolyte/electrolyte interface (*V*_E/P  _< 0 V). On the other hand, when *c* » *N*_fix_, the fixed charges in the polyelectrolyte are negligible, *c*^PSS^_+_ ≈ *c* and *V*_E/P_ ≈ 0 V.

This behavior resembles the Donnan equilibrium in semi-permeable membranes and the potential at the polyelectrolyte/electrolyte interface can be calculated as (see Supplementary Note 2)^25,46^:

6 $$V_{{\mathrm{E}}/{\mathrm{P}}} = - \frac{{k_{\mathrm{B}}T}}{q}{\mathrm{asinh}}\left( {\frac{{z_{{\mathrm{fix}}}N_{{\mathrm{fix}}}}}{{2zc}}} \right)$$

where *z* and *z*_fix_ are the number of charges of the ions dissolved into the electrolyte and fixed charge into the polyelectrolyte, respectively. Interestingly the model predicts that *V*_E/P_ shows a Nernstian behavior when *c* « *N*_fix_, while *V*_E/P_ ≈ 0 when *c* » *N*_fix_. Figure 3b (line) shows that *V*_E/P_ calculated with Eq. (6) accurately reproduces the measurements in the whole range of ion concentrations. By reproducing the measurements with the model we estimated a SO_3_^−^ concentration *N*_fix_ = 3.16 M (viz. about 1.9 10^21^ cm^−3^). The wide range of ion concentrations here assessed proves that the Nernst equation fails to describe *V*_E/P_ and hence the faradaic redox reactions can be disregarded. In addition, Supplementary Fig. 7 shows that Eq. (6) is able to explain the threshold voltage shift measured in OECTs exploiting electrolytes with different chemical nature, such as KCl, Ca(NO_3_)_2_, and Al_2_(SO_4_)_3_. This provides further insight on the strong dependence of the transistor performance on the chemical nature of the counterion used, as reported in refs. ^47,48^.

**Fig. 5:**
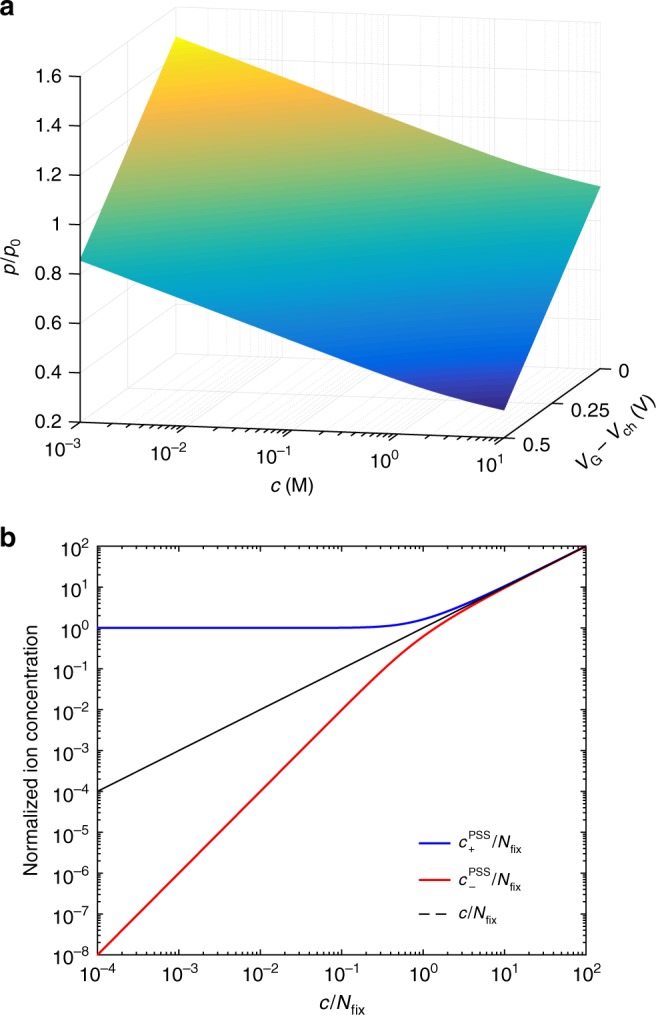
Hole and ion concentration in the OECT channel. **a** Hole concentration in the PEDOT:PSS channel normalized to the intrinsic hole concentration as a function of *c* and *V*_G_ − *V*_ch_. The hole concentration is calculated according to Eq. (7) at a channel coordinate *x* = *L*/2 when the OECT is biased at *V*_D_ = −0.1 V. **b** Mobile ion concentration in the electrolyte (black dashed line), and mobile cation (blue line) and anion (red line) concentration in the PSS polyelectrolyte normalized to the fixed charge concentration into the polymer *N*_fix_. The concentrations are calculated with Supplementary Equations (19), (20), (21) (see Supplementary Note 2)

Interestingly, Eqs. (4)–(6) show that the actual gating voltage (viz. *V*_G_ + *V*_SH_) depends on the electrolyte concentration (*V*_SH_ = *V*_G/E_ + *V*_E/P_). Combining this model with the approach proposed by Friedlein et al.^40^, the normalized hole concentration (*p*/*p*_0_) as a function of *c* results:

7 $$\frac{p}{{p_0}} = \frac{{V_{\mathrm{P}} - V_{\mathrm{G}} + V_{{\mathrm{ch}}}}}{{V_{\mathrm{P}}}} - \frac{{k_{\mathrm{B}}T}}{{qV_{\mathrm{P}}}}\left[ {\log \left( c \right) - {\mathrm{asinh}}\left( {\frac{{z_{{\mathrm{fix}}}N_{{\mathrm{fix}}}}}{{2zc}}} \right)} \right]$$

where *V*_ch_ is the potential along the polymeric channel. Figure 5a shows *p* as a function of *c* and *V*_G_. *p*/*p*_0_ linearly decreases with the logarithm of *c* with a slope equal to 147 10^−3^ dec^−1^ in the low range of *c* (10^−3^ M < *c* < 1 M) where *c* affects both *V*_E/P_ and *V*_G/E_, and 74 10^−3^ dec^−1^ in the high range of *c* (1 M < *c* < 5 M) where *V*_E/P_ vanishes and only *V*_G/E_ depends on *c*. Figure 5a also shows the dependence on *V*_G_ *–* *V*_ch_. In the whole range of *c*, *p/p*_0_ linearly decreases by increasing *V*_G_ since *C*_v_ is constant.

Figure 5b shows the mobile cation and anion concentrations in the PSS bulk (*c*^PSS^_+_ and *c*^PSS^_-_, respectively) as a function of the electrolyte ion concentration *c*. For the sake of generality, *c*^PSS^_+_, *c*^PSS^_-_, and *c* are normalized with respect to *N*_fix_. When *c* / *N*_fix_ « 1, *c*^PSS^_+_ is buffered to a constant value *c*^PSS^_+_ = *N*_fix_, while *c*^PSS^_−_ quadratically increases with *c*. In this case a concentration-dependent potential rises across the electrolyte/polymer interface. On the other hand, when *c / N*_fix_ » 1, the effect of the electrostatic interaction between the fixed charges and the mobile ions is negligible. It follows that the concentration of both anions and cations in the PSS phase is equal to *c* and a vanishing potential at the interface is obtained.

**Fig. 6:**
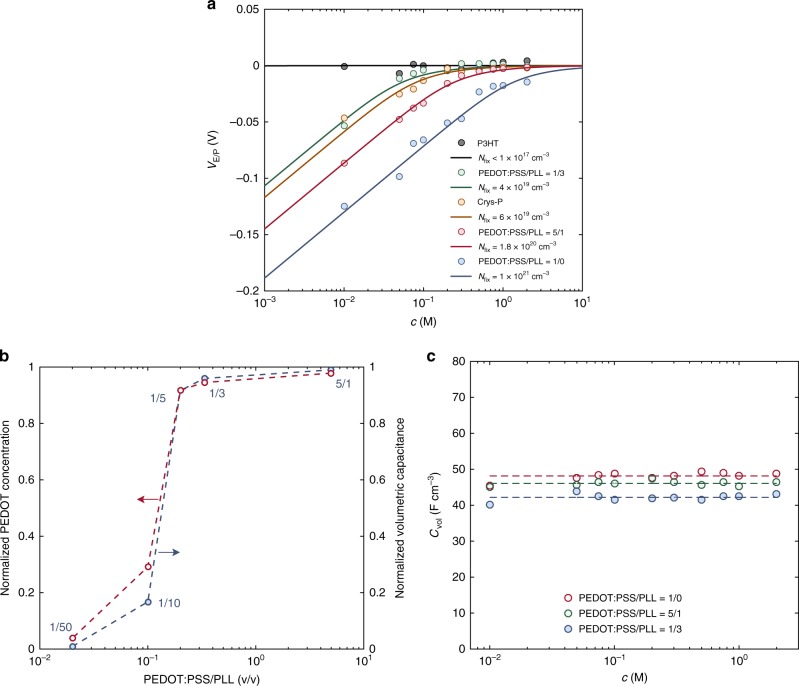
The effect of bulk and interface charge. **a** Measured (symbols) and calculated (solid lines, Eq. (6)) *V*_E/P_ as a function of *c* in OECTs with various polymeric channels. **b** PEDOT concentration (red symbols, left axis) and volumetric capacitance *C*_v_ (blue symbols, right axis) of PEDOT:PSS:PLL OECTs as a function of the PEDOT:PSS/PLL ratio. The *C*_v_ is normalized to the *C*_v_ of pristine PEDOT:PSS. Analogously, the PEDOT concentration is normalized to the PEDOT concentration of PEDOT:PSS OECTs. The dashed lines are guide for the eye. **c** Measured *C*_v_ as a function of *c* for OECTs with various PEDOT:PSS/PLL ratio (symbols). The dashed lines are the mean values

As a further confirmation, we fabricate OECTs including a positively charged polyelectrolyte in the channel. Poly-L-lysine (PLL) is added to the PEDOT:PSS dispersion by varying the ratio between PLL and PEDOT:PSS (details are provided in the Methods section). The electrical analyses (EIS and transfer characteristics) of the PEDOT:PSS:PLL OECTs are performed and the device parameters are extracted. Figure 6a shows the electrolyte/polymer potential *V*_E/P_ as a function of *c* when the PEDOT:PSS/PLL ratio is equal to 1/3, 5/1 and 1/0 (no PLL) v/v. The minimum *c* where *V*_E/P_ ≈ 0 V systematically lowers with increasing PLL concentration. In all cases the model (Eq. (6)) accurately reproduces the measurements in the whole range of ion concentrations and we found that the net negative fixed charge concentration in the PEDOT:PSS:PLL amounts to *N*_fix_^(5/1)^ *=* 1.8 10^20^ cm^−3^ (0.3 M) and *N*_fix_^(1/3*)*^ *=* 4 10^19^ cm^−3^ (66 10^−3^ M) when the PEDOT:PSS/PLL ratio is equal to 5/1 and 1/3 v/v, respectively. These results show that the potential at the electrolyte/polymer interface can be controlled by tuning the fixed charge concentration in the polymer. To investigate the generality of the model we extend the analysis to other materials, namely P3HT and Crystalized PEDOT:PSS (Crys-P)^49^, which are widely used for the fabrication of high performance OECTs^21,47,50^. Figure 6a shows that in the case of P3HT OECTs *V*_E/P_ is equal to 0 V in the whole concentration range explored, resulting in *N*_fix_^P3HT^ < 10^17^ cm^−3^ (10^−4^ M). This small fixed charge concentration is expected in the case of undoped P3HT^51–53^. Moreover, Fig. 6a shows that in the case of Crys-P the minimum *c* where *V*_E/P_ vanishes is shifted to lower *c* with respect to pristine PEDOT:PSS and we found *N*_fix_^Crys−P^ = 6 10^19^ cm^−3^. This provides quantitative evidence that in Crys-P the excess PSS (viz. bulk PSS) is chemically removed by sulfuric acid treatment. Interestingly, we found that *N*_fix_^Crys−P^ is almost equal to that obtained in PEDOT:PSS:PLL when the PEDOT:PSS/PLL ratio is 1/3 v/v, suggesting that in the case of PEDOT:PSS/PLL = 1/3 the bulk fixed charge is completely compensated.

### The role of the interface charge interaction

**Fig. 7:**
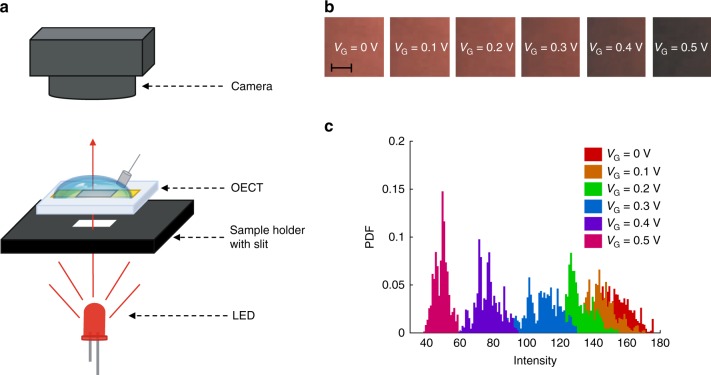
Electro-optical characterization of OECTs. **a** Schematic of the measurement setup. **b** 2D intensity maps measured on an OECT with PEDOT:PSS/PLL = 5/1 at *V*_G_ = [0, 0.1, 0.2, 0.3, 0.4, 0.5] V and *V*_D_ = *V*_S_ = 0 V. The scale bar is 50 µm. **c** Probability density function (PDF) of the 2D intensity maps measured on an OECT with PEDOT:PSS/PLL = 5/1 at various *V*_G_

To further assess the effect of the polyelectrolyte charges on the OECTs characteristics, we fabricate PEDOT:PSS:PLL OECTs with PEDOT:PSS/PLL ratio equal to 5/1, 1/3, 1/5, 1/10 and 1/50. Supplementary Figure 8a shows that at small PLL contents (viz. PEDOT:PSS/PLL larger than 1/3 v/v) the PEDOT is well dispersed and no visible polymer separation is obtained. By increasing the PLL content a clear separation of PEDOT from the dispersion shows up. This is confirmed by Supplementary Fig. 8b where, after the syringe withdrawal of the dispersion, no significant PEDOT residue is obtained at PEDOT:PSS/PLL ratios equal to 5/1 and 1/3 v/v, while PEDOT aggregates can be clearly seen at larger PLL concentrations. The formation of undispersed PEDOT is not surprising, considering that PEDOT is a water-insoluble polymer and the negative charges of the PSS are used as charge-balancing dopants to yield a water-soluble PEDOT:PSS complex^54^. It follows that the compensation of the negative fixed charges of the PSS located at the PEDOT/PSS interface by the positive charges of the PLL yields water-insoluble PEDOT aggregates. To quantitatively assess the PEDOT concentration in PEDOT:PSS:PLL OECTs we electro-optically investigate the composition of the deposited films. Figure 7a shows the experimental setup. We measure the optical transmittance at 620 nm^55^ and *V*_G_ ranging from 0 V to 0.5 V of devices with various amount of PLL. Figure 7b shows the images acquired in the case of an OECT with PEDOT:PSS/PLL = 5/1 and Fig. 7c shows the corresponding histogram of the red channel intensity at various *V*_G_. By applying the Beer-Lambert law, the measured optical intensity provides the ratio between the PEDOT concentration in PEDOT:PSS:PLL and pristine PEDOT:PSS. Figure 6b shows the PEDOT concentration in the PEDOT:PSS:PLL films as a function of the PEDOT:PSS/PLL ratio. When PEDOT:PSS/PLL ≥ 1/3 the PEDOT concentration into the PEDOT:PSS:PLL film is independent of the PLL content and equals to that of pristine PEDOT:PSS (without PLL), whereas further increasing the PLL content results in a reduction of the PEDOT content. Importantly, the electro-optical measurements show that the PEDOT doping state is not affected by PLL (Supplementary Note 3, Supplementary Fig. 9). We can conclude that small contents of PLL (PEDOT:PSS/PLL ≥ 1/3 v/v) result in a compensation of the fixed charges in the bulk of the polyelectrolyte with negligible effect on the fixed charges at the PEDOT/PSS interface. Besides, large amounts of PLL (PEDOT:PSS/PLL ≤ 1/5 v/v) result in a separation of PEDOT from the dispersion due to the compensation of the interfacial charges.

Taking advantage of this charge compensation effect, we investigate the volumetric capacitance as a function of the polyelectrolyte charge by performing EIS on PEDOT:PSS:PLL OECTs with various PEDOT:PSS/PLL ratios. Figure 6b shows that *C*_v_ of OECTs with small PLL contents (PEDOT:PSS/PLL > 1/5 v/v) is almost equal to the one of pristine PEDOT:PSS OECTs, *C*_v_ slightly decreases for devices with PEDOT:PSS/PLL ratio equal to 1/5, while it dramatically decreases at larger PLL contents. In the light of the previous analysis, small PLL contents affect only the bulk of the polymer without affecting the PEDOT:PSS interface. Therefore, we can conclude that *C*_v_ depends on the amount of PEDOT in the deposited film demonstrating that *C*_v_ is related to an electrostatic interaction at the semiconductor/(poly)electrolyte (PEDOT/PSS) interface. Figure 6c shows that the measured *C*_v_ is independent of the ion concentration. This behavior can be explained by considering the large concentration of fixed charge (about 1 M) at the PEDOT/PSS interface, which yields a negligible diffuse layer^42^.

### OECT based electronics

**Fig. 8:**
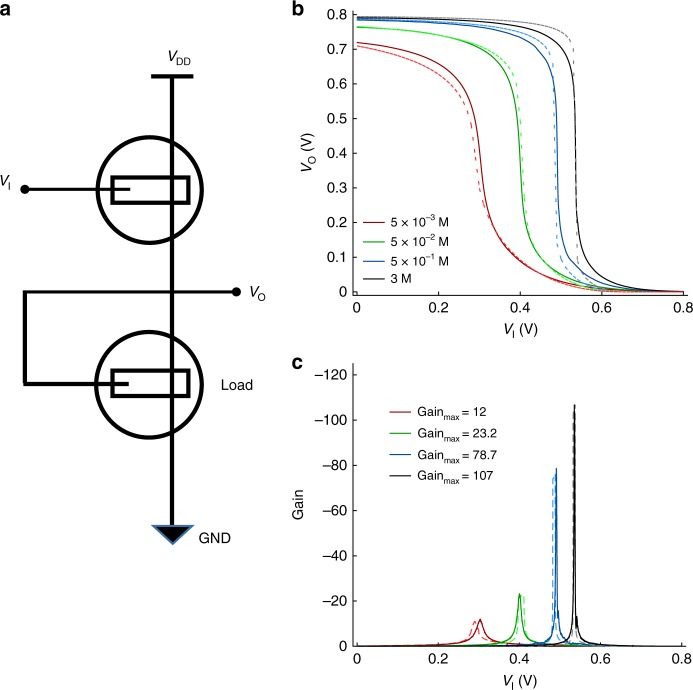
Unipolar OECT based inverter. **a** Schematic of the designed circuit. The driver geometries are *W* = 1000 µm, *L* = 500 µm, *t* = 210 nm, the load geometries are *W* = 1000 µm, *L* = 500 µm, *t* = 280 nm. Crys-P is used for the OECT channel, and details are provided in Supplementary Note 4. **b** Measured (solid line) and simulated (dashed lines) voltage transfer characteristics of the inverter. **c** Measured (solid line) and simulated (dashed lines) gain of the inverter

The understanding and quantification of the key mechanisms in OECTs, provide the rationale for the development of high-performance electronics. Here, as a relevant example, we demonstrate unipolar inverters. The inverter is a fundamental building block of any electronic circuit enabling the development of OECT-based integrated electronics and bioelectronics. Inverters are used both in digital circuits as logic gates and in analogue circuits as voltage amplifiers. Figure 8a shows the circuit schematic, which comprises two p-type OECTs connected in series. The input voltage *V*_I_ is applied to the gate of the Driver OECT while the Load OECT is operated as a zero-V_GS_ topology^56^. The OECT model (Supplementary Note 4, Supplementary Fig. 10) is implemented in a circuit simulator in order to predict the circuit operation.

The ion concentration of the Load OECT is exploited as a design parameter to maximize the key circuit figures of merit, namely the gain and noise margin, while the Driver ion concentration is 5 M. Figure 8b shows that the simulated (dashed lines) and measured (full lines) transfer characteristics are in agreement in the whole range of voltages and ion concentrations. When the input is low (e.g., *V*_I_ = GND) the source-gate voltage *V*_SG_ applied to the Driver OECT is large (*V*_SG_ = *V*_DD_ − *V*_I_) and, as a result, the output voltage *V*_OUT_ is close to *V*_DD_. By increasing *V*_I_, the pull-up becomes progressively weaker and a sharp transition of *V*_O_ from *V*_DD_ to GND is displayed when *V*_I_ reaches the trip-point voltage *V*_TP_. Further increasing *V*_I_ (*V*_I_ > *V*_TP_) reduces *V*_SG_ and *V*_O_ is close to GND. Figure 8b shows that the transfer characteristics are strongly affected by the ion concentration. More in detail, when *c* increases from 5 10^−3^ to 3 M *V*_TP_ shifts from *V*_I_ = 0.3 V to *V*_I_ = 0.54 V and the gain increases from 12 up to 107 (Fig. 8c), with a maximum noise margin equal to 0.33 V, i.e., 82% of the theoretical limit. This can be explained as follows. By increasing *c*, ion buffering yields a more negative threshold voltage of the Load transistor while, due to the interface charge, its capacitance is unaffected. As a result, *V*_TP_ increases since a larger *V*_I_ is required to switch *V*_O_ from the high to the low state. In addition, the reduced drain current of the Load transistor yields a larger gain, which is inversely proportional to the current flowing through the inverter. It is worth to note that for the sake of simplicity we varied only the Load electrolyte concentration, but similar considerations hold in the case that both the Load and the Driver concentrations are taken as design variables.

The proposed approach is compared with several inverters based on electrolyte-gated technologies in Table 1. The gain and noise margin of the designed inverter outperform those obtained by state-of-art electrolyte-gated inverters based on metal-oxides, carbon nanotubes, two-dimensional and organic materials^33,57–66^.

**Table 1:** Performance comparison

Transistor technology	Configuration	Material	Supply voltage (V)	Maximum gain (V/V)	Noise margin (%)	Refs
EGT	Unipolar	ZnO	2.0	8	65	^57^
EGT	Unipolar	IGZO	0.5	3.8	25	^58^
EGT	Complementary	P3HT, ZnO	1.0	18	20	^59^
EGCNT	Unipolar	Carbon nanotubes	0.8	2.4	25	^60^
EGCNT	Complementary	Carbon nanotubes	2.0	20	25	^61^
TT	Unipolar	MoS_2_	1.5	17	31	^62^
EGOFET	Unipolar	P3HT	1.0	7	67	^63^
EGOFET	Complementary	P(NDI2OD-T2), P(T_0_T_0_TT_16_)	1.0	17.5	70	^64^
EGOFET	Complementary	PNDIT2, pBTTT	2.0	2	60	^65^
EGOFET	Complementary	PNDISVS, pBTTT	0.8	3.6	25	^65^
EGOFET	Complementary	PCBM, P3HT	0.8	7	50	^65^
OECT	Unipolar	PEDOT:PSS	0.8	27	80	^33^
OECT	Complementary	BBL, P3CPT	0.6	12	67	^66^
OECT	Unipolar	Crys-P	0.8	107	82	This work

The table compares several inverters fabricated with electrolyte-gated transistor technologies by considering the supply voltage, maximum gain, and percentage noise margin. The gain is calculated as d*V*_O_/d*V*_I_, the noise margin (NM) is calculated as reported in ref. ^33^, and the percentage noise margin (NM_%_) is NM normalized to the supply voltage NM_%_ = NM2 *V*_DD_^−1^

## Discussion

In summary, we provide experimental evidence of the electrostatic nature of the ionic-electronic interaction in OECTs. We perform both electrical *I*_D_-*V*_G_ characteristics and electrochemical impedance spectroscopies on OECTs by exploring a wide range of channel thicknesses, polymer formulations and electrolyte ion concentrations. We show that to understand the OECT operation it is crucial to consider the bulk and interface electrostatic charge compensation taking place in the transistor channel. More in detail, the fixed charges in the bulk of the polyelectrolyte are electrostatically compensated by the mobile ions provided by the electrolyte. This results in an ion buffering in the polymeric channel and, in turn, in a concentration-dependent voltage drop at the polyelectrolyte/electrolyte interface. The fixed charges close to the semiconductor are electrostatically compensated by the electronic charges accumulated at the semiconductor/polyelectrolyte interface. This bulk uptake of ions results in a capacitance distributed in the volume of the polymer and independent of the electrolyte ion concentration. The ion concentration dependent transfer characteristics are quantitatively explained. The analysis is extended to OECTs with different polymers, proving its generality. We show that a large set of measurements in a wide range of experimental conditions can be consistently explained without invoking faradaic reactions. The impact of our analysis on the design of OECT based circuits is demonstrated by the design of unipolar inverters with tunable performances, showing the highest gain and noise margin ever reported for this class of circuits.

Prospectively, our study opens opportunities for several applications and research directions. Our work provides both fundamental knowledge and experimental demonstration of OECTs with tailored ionic response. This finds application for future development of integrated bioelectronics^67^, electronic circuits, ion sensors^16,17^ and neuromorphic circuits. For example, in neuromorphic applications a wide range of ion concentration can be exploited as a global regulation parameter to emulate the homeoplasticity phenomena of neural environments^12,68^. Our work enables the design of neuromorphic circuits with synapse-specific response, typical of biological neural networks^69^. Finally, the demonstration of an ion concentration-independent volumetric capacitance is crucial for in vivo applications, as the ionic concentration of the body fluid may vary from person to person and between the different body fluids^34^, thus requiring OECTs with a transconductance independent of the ionic environment. Our findings provide important design rules for the rational design of devices with enhanced performance and application-specific functionalities, opening opportunities for the next generation OECT-based electronic, bioelectronics and neuromorphic computing.

## Methods

### PEDOT:PSS and PEDOT:PSS:PLL OECT fabrication

100 nm thick Au source/drain contacts are sputtered through a shadow mask on a polyethylene substrate. An adhesion layer of 15 nm of Ti-W is previously deposited to enhance Au adhesion. Polyethylene substrates are cleaned with DI water, dried with N_2_ and treated with oxygen plasma to promote PEDOT:PSS adhesion. An Epson XP-215 is used for the printing process, filling a cartridge with either PEDOT:PSS or PEDOT:PSS:PLL. PEDOT:PSS (PH-500 from Heraeus Clevios GmbH) is mixed with 5 vol% ethylene glycol, 1 vol% 3-glycidoxypropyl-trimethoxysilane, 0.25 vol% dodecyl benzene sulfonic acid as conductivity enhancer, crosslinker and wetting agent, respectively. PEDOT:PSS:PLL is obtained by adding to PEDOT:PSS a PLL solution (0.1 % w/v in H_2_O) at various volume ratios. All the reagents were purchased from Sigma-Aldrich. The dispersion is filtered through a syringe filter (regenerated cellulose, pore size 0.2 µm) prior to deposition. The tuning of the film thickness is obtained by printing multiple stacked layers. A minimum of 4 layers is necessary to obtain a continuous conductive film. The thickness of the polymeric film obtained by varying the number of printed layers is measured with a stylus profilometer Bruker Dektak XT. A soft bake (1 min at 50 °C) is performed between the printing of each layer to avoid the spread of the printed polymer. The films are baked at 100 °C for 1 h and immersed in deionized water to remove any excess low molecular weight compounds.

### Crys-P OECT fabrication

We fabricate Crys-P OECTs following the fabrication process reported in ref. ^50^. In brief, PEDOT:PSS (PH-500 from Heraeus Clevios GmbH) is filtered (regenerated cellulose, pore size 0.2 µm) and spin coated on quartz-coated substrates with sputtered gold source and drain electrodes (*W* = 1000 µm, *L* = 100 µm). The deposited films are annealed at 120 °C for 15 min and then immersed in a bath of concentrated H_2_SO_4_ (>95%, Sigma Aldrich) for 15 min, thoroughly rinsed with deionized water, and dried at 120 °C for 15 min.

### P3HT OECT fabrication

We fabricate P3HT OECTs following the fabrication process reported in ref. ^21^. Briefly, 25 mg ml^−1^ of regioregular P3HT (Ossila M105) are dissolved overnight at 80 °C in dichlorobenzene. The solution is filtered through a 0.45 µm PTFE filter and spin coated (3 s at 500 r.p.m., 60 s at 1000 r.p.m., 10 s at 5000 r.p.m. from ∼45 °C solution) on Si-SiO_2_ substrates with gold interdigitated source and drain electrodes (*W* = 1500 µm, *L* = 5 µm).

### Electrical characterization

Transfer and output characteristics are measured with a Keithley 2636 A SourceMeter Unit. A solution of NaCl in DI water at several concentrations is used as electrolyte and an Ag/AgCl pellet (Warner Instruments) is used as gate electrode. Transfer characteristics are measured by sweeping the gate voltage, with the source electrode grounded and the drain electrode polarized at a constant potential. Output characteristics are measured by sweeping the drain voltage, with the source electrode grounded and the gate electrode polarized at a constant potential. The electrical characteristics of the devices are measured with a constant sweep rate of 18 mV/s, which allows the device to reach the steady state operation regime. Electrochemical Impedance Spectroscopy measurements are performed using a National Instruments PXI-1042 system, equipped with a PXI-5112 oscilloscope, a PXI-5421 arbitrary wave-function generator and custom Labview software for the control of the system. A three-electrode configuration is adopted. The drain and source electrodes are shortened together and the OECT channel acted as the working electrode. Two Ag/AgCl pellets serve as reference and counter electrodes. The inverter characteristics are measured with a Keithley 2636 A Source Meter Unit. The input voltage is swept from 0 V to 0.8 V with a voltage step of 0.3 mV and at supply voltage of 0.8 V.

### Electrical and optical characterization

The OECT is placed on an opaque sample holder with the channel in correspondence of a slit. A droplet of 10^−1^ M NaCl solution is placed on top of the transistor and an Ag/AgCl gate is dipped into the solution. The source and drain electrodes are shortened and *V*_D_ = *V*_S_ = 0 V, while the gate voltage is biased in the range 0 to 0.5 V. The light from a red light emitting diode (Superlight, LED emission peak at wavelength 620 nm) placed below the sample holder is conveyed at the transistor channel through the holder slit and the transmitted light is captured by a camera (Dino-Lite AM4023CT, raw acquisition) on the opposite side of the sample. The measurements are performed in a dark room. The 2D transmitted intensity map was acquired on a scale from 0 to 255 taking care that no pixel saturated at 0 or 255.

## Supplementary Information

Supplementary Information

## Data Availability

The data that support the findings of this study are available from the corresponding author on reasonable request.
