# Supplementary material for: Ion buffering and interface charge enable high performance electronics with organic electrochemical transistors
Source: Nat Commun. 2019 Jul 10;10:3044. doi: 10.1038/s41467-019-11073-4 (PMC6620344; doi:10.1038/s41467-019-11073-4)
Supplement: Supplementary file 1 — Supplementary Information [file 41467_2019_11073_MOESM1_ESM.pdf]

Supplementary Information

Ion buffering and interface charge enable high performance electronics with organic electrochemical transistors

Paolo Romele[1], Matteo Ghittorelli[1], Zsolt Miklós Kovács-Vajna[1], & Fabrizio Torricelli[1]

[1]University of Brescia, Department of Information Engineering, via Branze 38, 25123 Brescia, Italy.

Correspondence and requests for materials should be addressed to F.T. (email: fabrizio.torricelli@unibs.it)

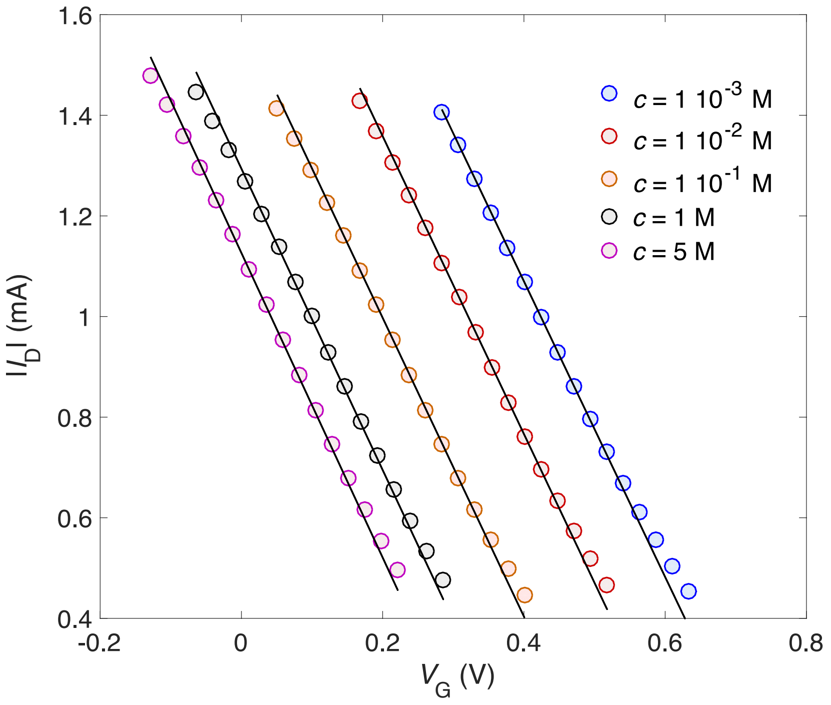

Supplementary Figure 1 OECTs electrical characteristics. Transfer characteristics measured in the linear region (V_D = -0.1 V) at several electrolyte concentrations (symbols) and fit with drain current model[1,2] (solid line) in the case of an OECT with t = 2300 nm. The transfer characteristics are measured in a narrow range of V_G to avoid possible device degradation[3,4].

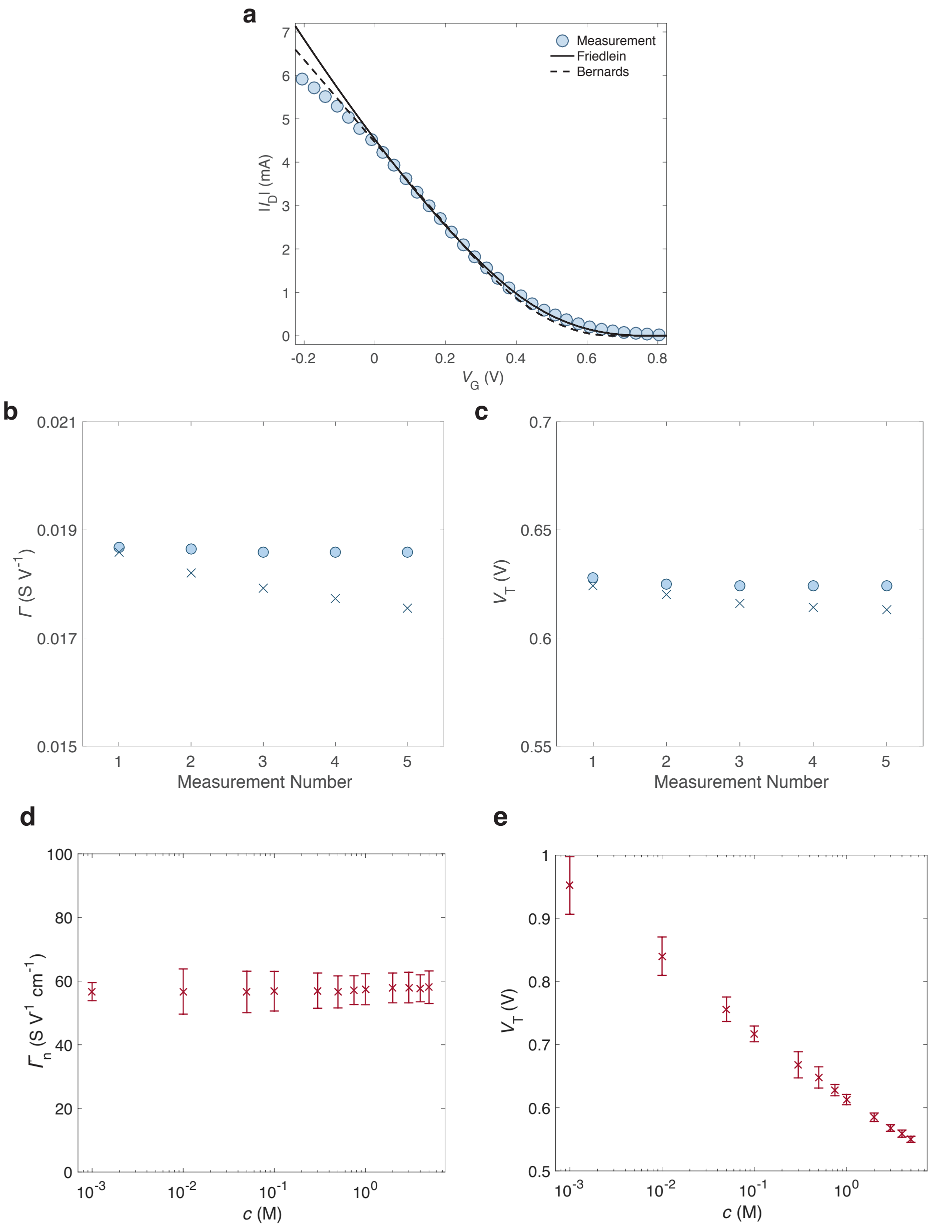

Supplementary Figure 2 Modelling validation. a Transfer characteristic of an OECT with t = 2095 nm, at V_D = -0.4 V and $c = 10^{-2}$ M measured (symbols) in an extended range of V_G (-0.2 V ≤ V_G ≤ 0.8 V). The dashed line is the fit with the Bernards-Malliaras model[1] and the full line is the fit with the Friedlein et al. model[5] (Supplementary Note 1). Both the models accurately describe the linear regime (0 V ≤ V_G ≤ 0.3 V), while the Friedlein et al. model provides superior performance at V_G > 0.3 V. **b-c** physical parameters Γ and V_T as a function of the number of repeated measurements. The parameters are extracted from I_D-V_G measured in a narrow range (0.15 V ≤ V_G ≤ 0.5 V, circles) and wide range (-0.2 V ≤ V_G ≤ 0.8 V, crosses) with the Bernards-Malliaras model. The measurements in a narrow range provide physical parameters consistent with those obtained by modeling the measurements in a wide range (circles and crosses overlapped at the first measurement), while providing excellent stability of the extracted parameters over repeated measurements. **d-e** Normalized conductivity $\Gamma_n = \Gamma L W^{-1} t^{-1}$ and threshold voltage V_T as a function of the ion concentration extracted with the Friedlein model. Crosses are the mean value and error bars are the standard deviation calculated by modeling ten OECTs at various thicknesses.

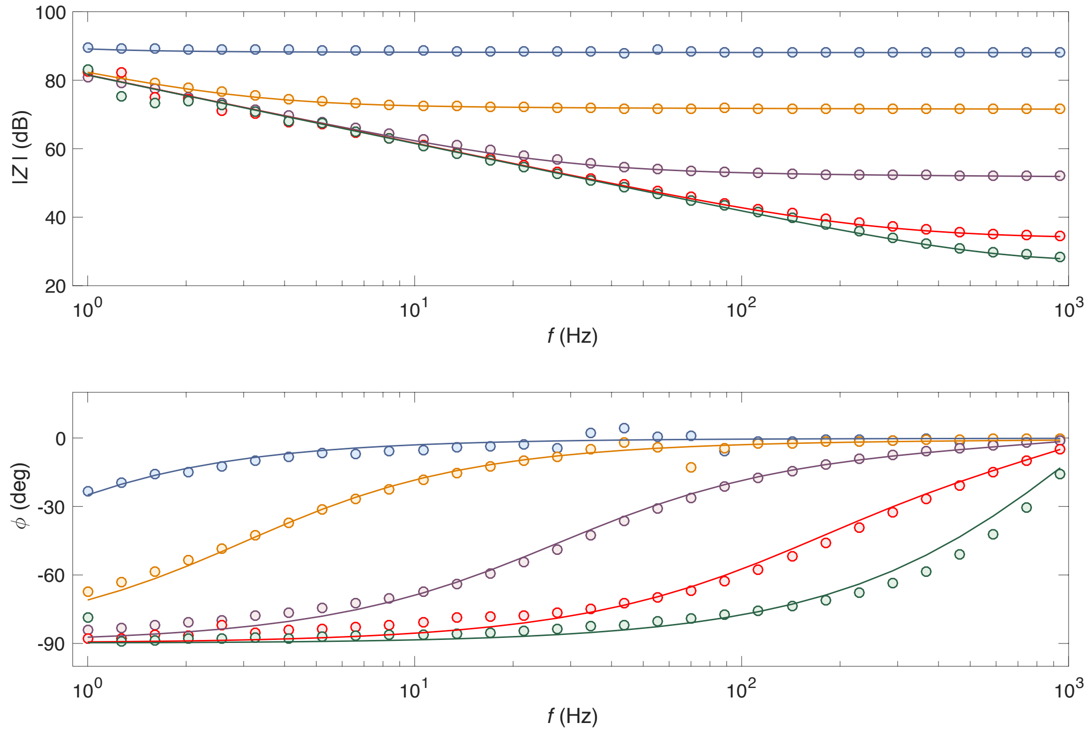

Supplementary Figure 3 OECT impedance spectra. Impedance spectra as a function of ion concentration c = [10^{-3}, 10^{-2}, 10^{-1}, 10^{0}, 5] M. The OECT geometries are W = 1000 µm, L = 500 µm, and t = 500 nm. We reproduced the measurements (symbols) with the Randles circuit model (full line) as described in Ref. [6] and we found R_p > 5 MΩ for all the ion concentrations, R_s is proportional to the inverse of the ion concentration, in agreement with the Debye-Hückel-Onsager theory[7], and the volumetric capacitance is independent of the ion concentration and results C_v = 44.3 ± 0.6 F cm^{-3}.

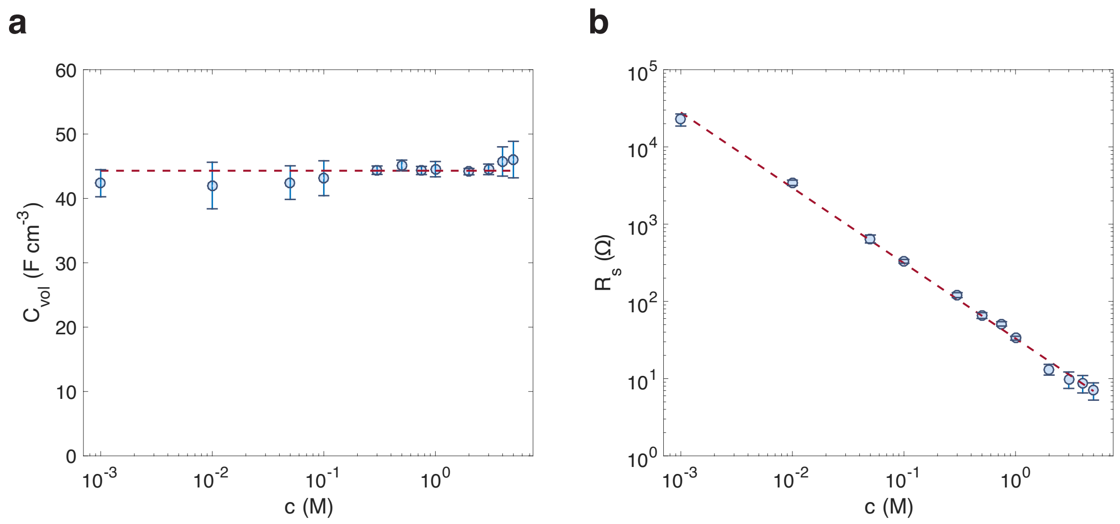

Supplementary Figure 4 EIS parameters. a Volumetric capacitance C_v as a function of c. Circles are the mean value and error bars are the standard deviation calculated by modelling ten OECTs with various thicknesses. The red dashed line is the overall mean value. **b** Mean value and standard deviation of R_s as a function of c (symbols). The red dashed line is the linear least square approximation in log-log scale.

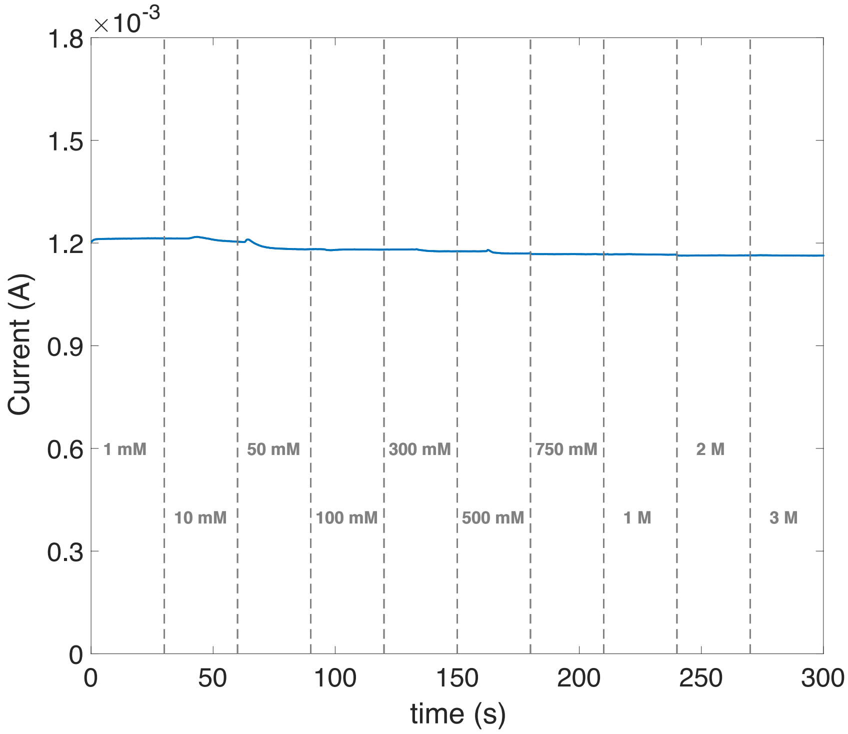

Supplementary Figure 5 Conductivity as a function of the ion concentration. The current flowing through a PEDOT:PSS OECT exposed to several electrolyte concentrations is measured without immersing the gate electrode into the electrolyte. The voltage applied between the source and drain electrodes is 0.1 V. The device is initially exposed to a 10^{-3} M NaCl aqueous solution and the NaCl concentration is increased every 30 seconds. The maximum electrolyte concentration is equal to 3 M.

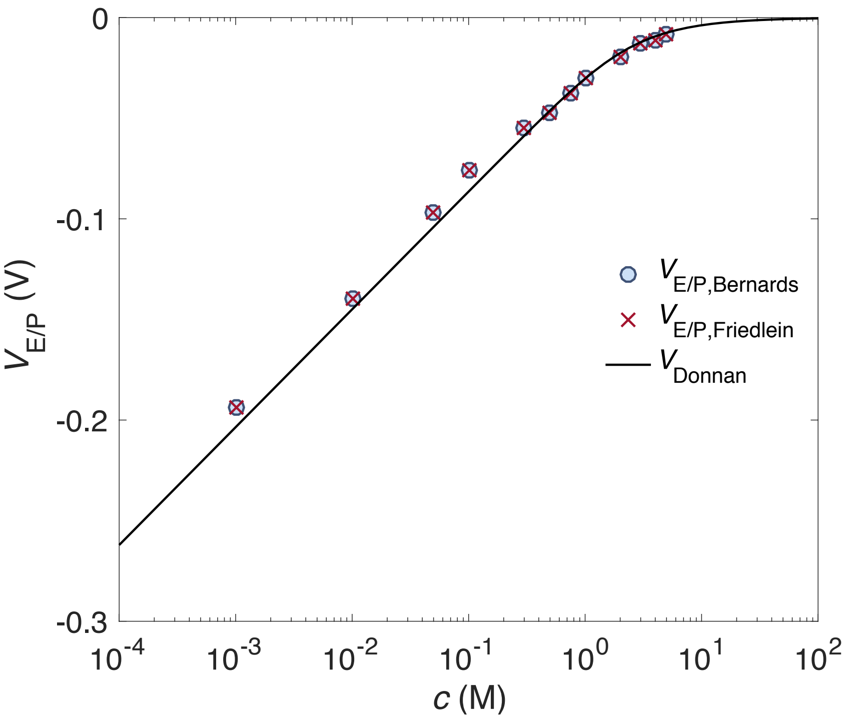

Supplementary Figure 6 Voltage at the electrolyte/polymer interface as a function of the ion concentration. Symbols are the measurements and solid line is calculated with Eq. (6). Circles and crosses are obtained with the Bernards-Malliaras model[1] and Friedlein et al. model[5], respectively. The two models provide the same $V_{E/P}$. This is expected because the variation of the ion concentration results in a rigid shift of the transfer characteristics, and this is described as a threshold voltage shift in both the models.

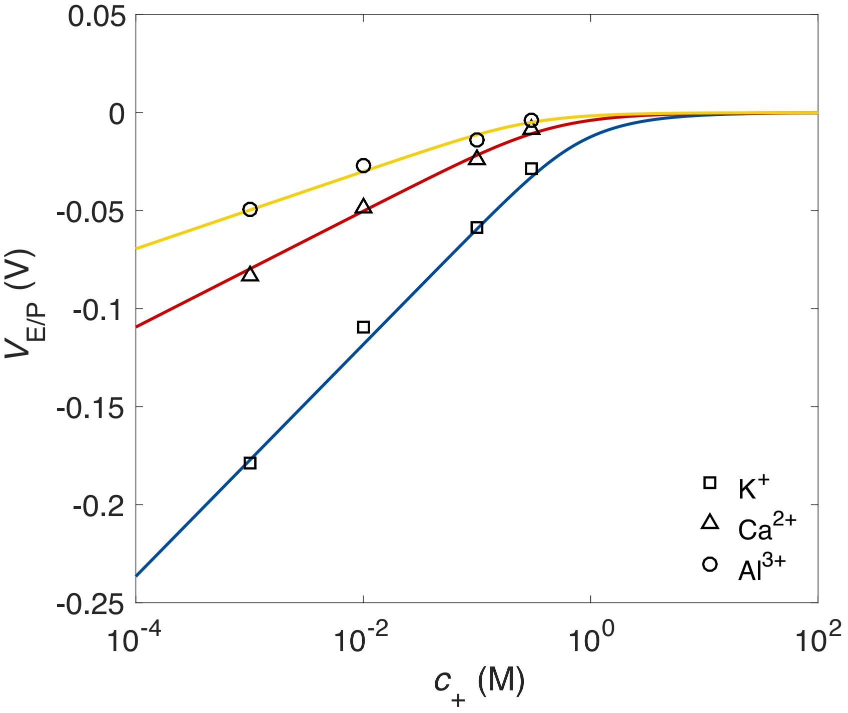

Supplementary Figure 7 Cationic specie influence on the potential at the electrolyte/polymer interface. $V_{E/P}$ as a function of the electrolyte concentration is reported in the case of KCl where $z_+ = 1$, $z_- = 1$, $c_+ = c_-$, $Ca(NO_3)_2$ where $z_+ = 2$, $z_- = 1$, $c_- = 2c_+$, and $Al_2(SO_4)_3$ where $z_+ = 3$, $z_- = 2$, $c_- = 3/2c_+$. Data (symbols) are taken from Ref. [8], while solid lines are $V_{E/P}$ calculated with Supplementary Equation (21) in Supplementary Note 2. The slope of $V_{E/P}$ in the low concentration range scales with z_+^{-1}, viz. 59, 30, 20 mV dec^{-1} when $z_+ = 1, 2, 3$ respectively, while the transition from logarithmic to constant characteristic (viz. minimum c where $V_{E/P} \approx 0$ V) shifts to lower c by increasing z_+.

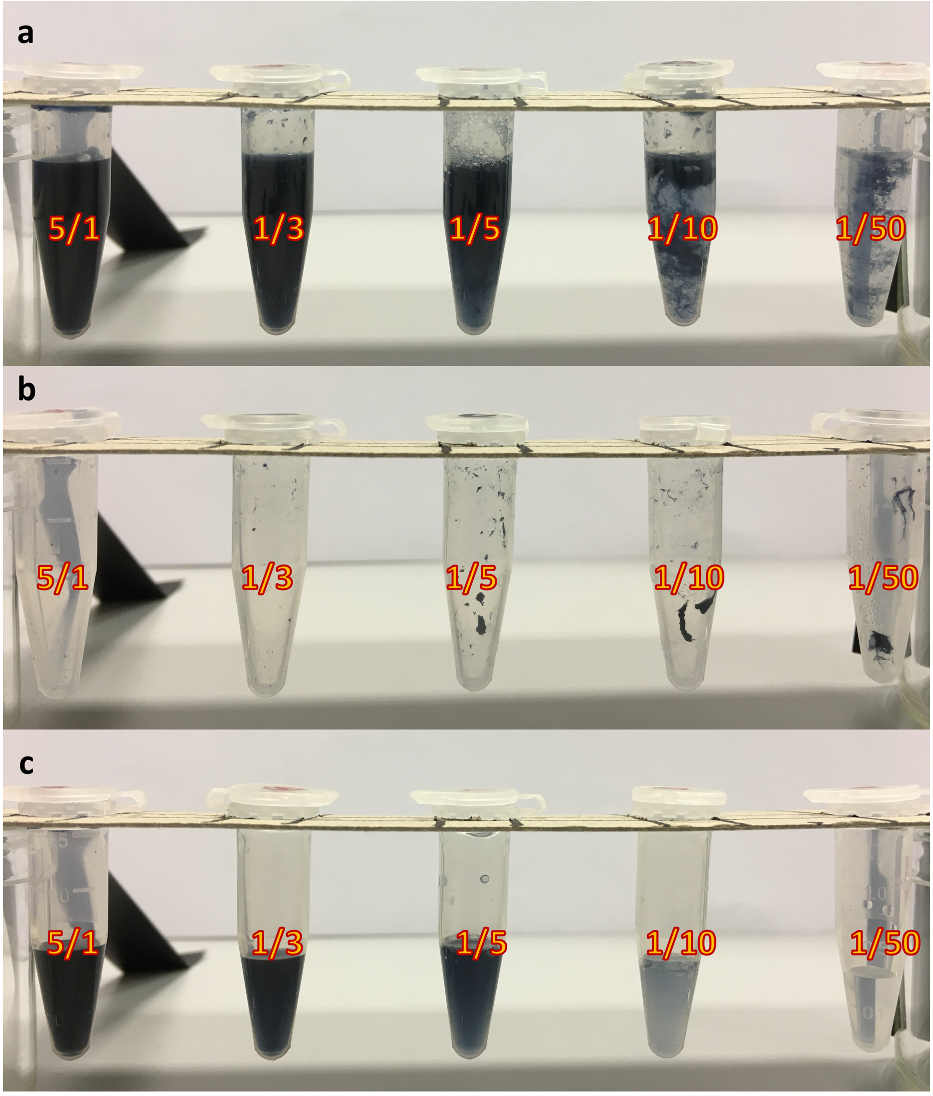

Supplementary Figure 8 Evaluation of the effect of PLL on the PEDOT:PSS dispersion. a PLL water solution is added to the PEDOT:PSS dispersion at PEDOT:PSS/PLL ratios 5/1, 1/3, 1/5, 1/10, and 1/50 v/v. **b** The dispersion is withdrawn by a syringe (21G needle). **c** The withdrawn dispersion is filtered through a filter (pore size 0.2 µm) and aggregates of undispersed PEDOT are eventually removed. The filtered dispersion is used for the fabrication of PEDOT:PSS:PLL OECTs.

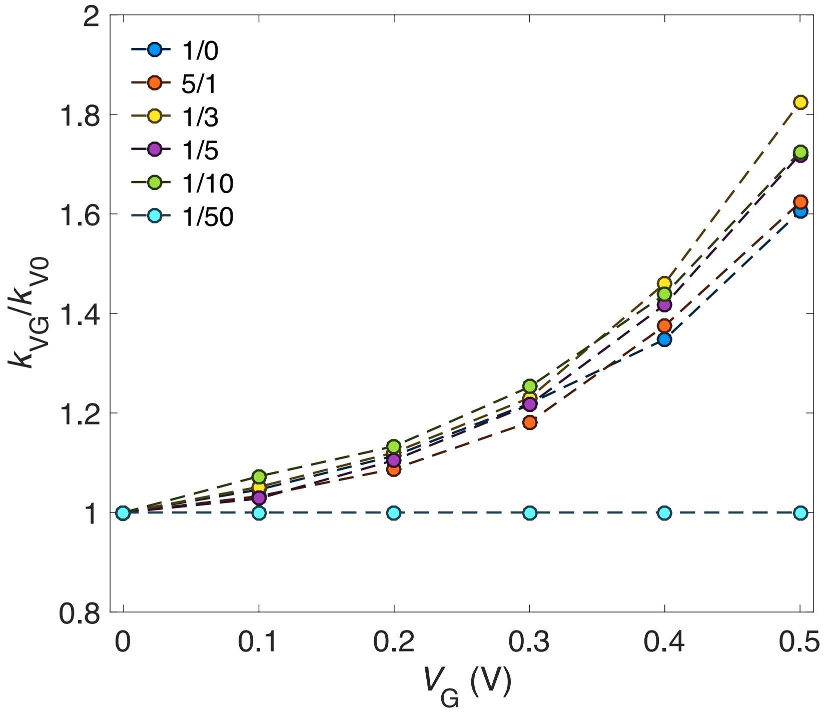

Supplementary Figure 9 PEDOT:PSS/PLL ratio effect on the molar attenuation coefficient. The molar attenuation coefficient at 620 nm is measured as a function of the gate voltage for OECTs with several PEDOT:PSS/PLL contents (1/0, 5/1, 1/3, 1/5, 1/10, 1/50). Values are normalized to the molar attenuation coefficient at 620 nm when V_G = 0 V.

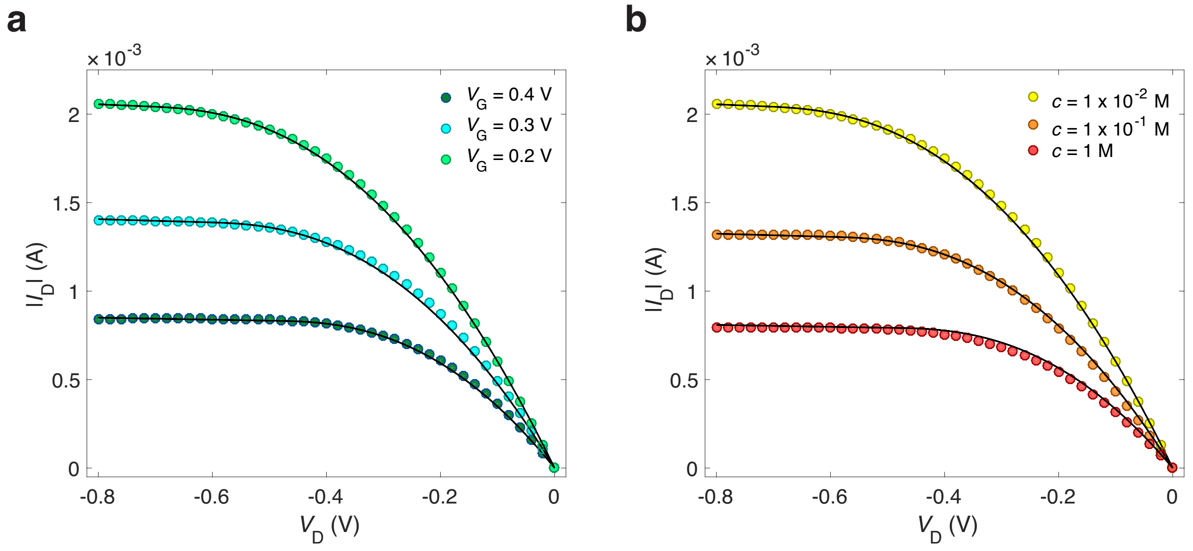

Supplementary Figure 10 Measured (symbols) and fitted (solid lines) output characteristics of OECTs. a Output characteristics measured at several gate bias, the electrolyte concentration is 1 x 10^{-2} M. **b** Output characteristics measured using several gate electrolyte concentrations, the gate bias is 0.2 V. The fitting is performed by using the model in Supplementary Note 4.

Supplementary Notes

Supplementary Note 1: Friedlein model

Friedlein et al.[5] described the drain current as follows:

$$I_\mathrm{D} = \frac{\Gamma}{\gamma} V_\mathrm{P}^{2-\gamma}[(V_\mathrm{T} - V_\mathrm{G} + V_\mathrm{D})^\gamma - (V_\mathrm{T} - V_\mathrm{G} + V_\mathrm{S})^\gamma] \quad \text{when } V_\mathrm{T} - V_\mathrm{G} \geq -V_\mathrm{D} \quad (1)$$

$$I_\mathrm{D} = -\frac{\Gamma}{\gamma} V_\mathrm{P}^{2-\gamma}(V_\mathrm{T} - V_\mathrm{G} + V_\mathrm{S})^\gamma \quad \text{when } V_T - V_G < -V_D \quad (2)$$

where

$$\Gamma = \frac{Wt}{L}\mu C_\mathrm{v} \quad (3)$$

$$\gamma = \frac{E_0}{k_\mathrm{B}T} + 1 \quad (4)$$

$$V_\mathrm{T} = V_\mathrm{P} - V_\mathrm{SH} \quad (5)$$

W, *L*, *t*, are the channel width, length and thickness, respectively, μ is the hole mobility, C_v is the volumetric capacitance, E_0 is the disorder parameter describing the energetic width of the tail of the density of states, k_B is the Boltzmann constant, *T* is the temperature, V_T is the threshold voltage, $V_\mathrm{P} = q\ p_0\ C_\mathrm{v}^{-1}$ is the pinch-off voltage, *q* is the elementary charge, p_0 is the intrinsic doping of the semiconductor, and V_SH accounts for the voltage shift as a function of the ion concentration *c*. It is worth noting that V_SH can be attributed to both the gate/electrolyte and electrolyte/polymer interfaces and reads: $V_\mathrm{SH} = V_\mathrm{G/E} + V_\mathrm{E/P}$.

According to Supplementary Equations (1)-(5), the Friedlein et al. model depends on the applied voltages (V_G, V_D, V_S) and on the OECT physical and geometrical parameters (Γ, V_T, γ). Compared to the Bernards-Malliaras model the Friedlein et al. model has an additional parameter, namely γ, which accounts for the energy disorder.

We further extended the analysis by systematically fitting the measurements of OECTs with several thicknesses and ion concentrations with the Friedlein et al. model. Supplementary Figures 2d,e show the extracted Γ normalized to the geometrical parameters, viz. $\Gamma_\mathrm{n} = \Gamma\ L\ W^{-1}\ t^{-1}$, and V_T. It is worth to note that V_T provided by the Friedlein et al. model is slightly different with respect to that obtained with the Bernards-Malliaras model due to the different extracted p_0. The other parameters of the model, namely V_P and γ, are the very same for all the OECTs and result V_P = 0.8 V and γ = 2.4. The comparison between the polyelectrolyte/electrolyte interface potential $V_\mathrm{E/P}$ obtained with the Bernards-Malliaras model (circles) and the Friedlein et al. model (crosses) is shown in Supplementary Figure 6

Supplementary Note 2: The electrolyte/polymer interface potential model

The Poisson Equation coupled with the Nernst-Planck equation allows to estimate the potential distribution and the ionic concentrations in the polyelectrolyte/electrolyte structure.

The Poisson equation reads:

$$\nabla \cdot \mathbf{D} = \rho \tag{6}$$

where D is the electric displacement field and ρ is the electric charge density. Assuming that the charge distribution and the electric potential are uniform in the plane parallel to the polyelectrolyte/electrolyte interface, the problem can be studied as a mono-dimensional problem along the x direction normal to the interface. Given $D_x = \varepsilon E$, where E is the electric field and ε is the permittivity of the medium, Supplementary Equation (6) can be written as:

$$\frac{\partial \varepsilon E}{\partial x} = q(z_+ c_+ - z_- c_- - z_{\text{fix}} N_{\text{fix}}) \tag{7}$$

where c_+, c_- and N_{fix} are the mobile cation, mobile anion and fixed charge concentrations, respectively, and z_+, z_- and z_{fix} are the number of charges (viz. absolute value of valency). By remembering that $E = - \partial \varphi / \partial x$, where φ is the electric potential, it follows:

$$\frac{\partial^2 \varphi}{\partial x^2} = -\frac{q}{\varepsilon}(z_+ c_+ - z_- c_- - z_{\text{fix}} N_{\text{fix}}) \tag{8}$$

According to the Nernst-Planck equation, the flux of ions can be written as:

$$j_- = z_- q \mu_{\text{c}_-} c_- E - z_- q D_{\text{c}_-} \frac{\partial c_-}{\partial x} \tag{9}$$

$$j_+ = z_+ q \mu_{\text{c}_+} c_+ E + z_+ q D_{\text{c}_+} \frac{\partial c_+}{\partial x} \tag{10}$$

where j_- and j_+ are the anionic and cationic charge fluxes along the direction normal to the polymer/electrolyte interface (x direction), respectively, and $\mu_{\text{c-}}$ ($\mu_{\text{c+}}$) and $D_{\text{c-}}$ ($D_{\text{c+}}$) are the electrophoretic mobility and the diffusivity coefficient of the considered anion (cation), respectively. The diffusivity coefficient and the electrophoretic mobility are related by means of the Nernst-Einstein equation:

$$D_{\text{c}_-} = \frac{k_{\text{B}} T}{z_- q} \mu_{\text{c}_-} \tag{11}$$

$$D_{\text{c}_+} = \frac{k_{\text{B}} T}{z_+ q} \mu_{\text{c}_+} \tag{12}$$

where k_{B} is the Boltzmann constant and T is the temperature. Considering the Nernst-Planck equation at the equilibrium ($j = 0$), Supplementary Equations (9) and (10) can be rewritten as:

$$q\mu_{c\_}c_{-}\left(\frac{k_{\mathrm{B}}T}{qc_{-}}\frac{\partial c_{-}}{\partial x}+z_{-}\frac{\partial\varphi}{\partial x}\right)=0 \quad (13)$$

$$q\mu_{c_{+}}c_{+}\left(\frac{k_{\mathrm{B}}T}{qc_{+}}\frac{\partial c_{+}}{\partial x}-z_{+}\frac{\partial\varphi}{\partial x}\right)=0 \quad (14)$$

and hence:

$$q\mu_{c\_}c_{-}\frac{\partial\left(\frac{k_{\mathrm{B}}T}{q}ln(c_{-})+z_{-}\varphi\right)}{\partial x}=0 \quad (15)$$

$$q\mu_{c_{+}}c_{+}\frac{\partial\left(\frac{k_{\mathrm{B}}T}{q}ln(c_{+})-z_{+}\varphi\right)}{\partial x}=0 \quad (16)$$

It follows from Supplementary Equations (15) and (16):

$$\ln(c_{-})=-\frac{q}{k_{\mathrm{B}}T}(z_{-}\varphi+A) \quad (17)$$

$$\ln(c_{+})=\frac{q}{k_{\mathrm{B}}T}(z_{+}\varphi+B) \quad (18)$$

where A, B are constant potential values and the anion and cation concentrations result:

$$c_{-}=c_{-,0}\mathrm{e}^{-\frac{z_{-}q}{k_{\mathrm{B}}T}\varphi} \quad (19)$$

$$c_{+}=c_{+,0}\mathrm{e}^{\frac{z_{+}q}{k_{\mathrm{B}}T}\varphi} \quad (20)$$

where $c_{-,0}$ = exp[-(k_{B} T / q) A], $c_{+,0}$ = exp[(k_{B} T / q) B] are constant values. $c_{-,0}$ and $c_{+,0}$ represent the anion and cation concentration in the bulk of the electrolyte. Combining Supplementary Equations (17) and (18) with Supplementary Equation (8) results:

$$\frac{\partial^{2}\varphi}{\partial x^{2}}=-\frac{q}{\varepsilon}\left(c_{+}z_{+}\mathrm{e}^{\frac{z_{+}q}{k_{\mathrm{B}}T}\varphi}-c_{-}z_{-}\mathrm{e}^{-\frac{z_{-}q}{k_{\mathrm{B}}T}\varphi}-z_{\mathrm{fix}}N_{\mathrm{fix}}\right) \quad (21)$$

The potential in the polyelectrolyte/electrolyte structure can be calculated by numerically solving Supplementary Equation (21). In the case that c_{+} = c_{-} = c and z_{+} = z_{-} = z, the potential at the polyelectrolyte/electrolyte interface can be analytically calculated as $V_{\mathrm{E/P}}$ = φ_{bulk} - $\varphi_{\mathrm{polymer}}$ and results:

$$V_{\mathrm{E/P}}=-\frac{k_{\mathrm{B}}T}{zq}\operatorname{asinh}\left(\frac{z_{\mathrm{fix}}N_{\mathrm{fix}}}{2zc}\right) \quad (22)$$

Supplementary Note 3: Optical investigation of PEDOT:PSS:PLL films

PEDOT:PSS:PLL OECTs with various amount of PLL are measured at V_G = [0, 0.1, 0.2, 0.3, 0.4, 0.5] V and the 2D map of the transmitted intensity is acquired. Then, the overall transmitted intensity is calculated as the mean value of the measured probability density function (PDF).

In order to quantify the PEDOT concentration of the PEDOT:PSS:PLL devices we measured the transmitted intensity of the samples with various amount of PLL at the same gate voltage (V_G = 0 V). By applying the Beer-Lambert law, the ratio between the transmitted intensity of a sample with a certain amount of PLL and the sample without PLL results:

$$\frac{I_{\mathrm{x}}}{I_{1/0}} = \frac{\exp(-kc_{\mathrm{x}}t_{\mathrm{x}})}{\exp(-kc_{1/0}t_{1/0})} \tag{23}$$

where I_x is the transmitted intensity measured in the case of PEDOT:PSS/PLL at x = [5/1, 1/3, 1/5, 1/10, 1/50], $I_{1/0}$ is the transmitted intensity measured with pristine PEDOT:PSS (viz. x = 1/0), t is the thickness of the sample, k is the molar attenuation coefficient of PEDOT, c_x is the molar concentration of PEDOT in the sample, and (k $c_{1/0}$) = 0.76 10^{-3} nm^{-1} [9]. The ratio between the PEDOT concentration in PEDOT:PSS:PLL samples and pristine PEDOT:PSS ($c_{1/0}$) can be calculated with Supplementary Equation (23) and results:

$$\frac{c_{\mathrm{x}}}{c_{1/0}} = \frac{\log\left(\frac{I_{1/0}}{I_{\mathrm{x}}}\right)}{kc_{1/0}t_{1/0}} + \frac{t_{\mathrm{x}}}{t_{1/0}} \tag{24}$$

Fig. 6b shows the PEDOT concentration in the PEDOT:PSS:PLL film normalized to that in the PEDOT:PSS film (without PLL) as a function of the PEDOT:PSS/PLL ratio. This analysis quantitatively agrees with the measured normalized volumetric capacitance, corroborates the qualitative analysis showed in the Supplementary Figure 8 and confirms that the volumetric capacitance in PEDOT:PSS(:PLL) OECTs arises from an electrostatic interaction at the semiconductor/(poly)electrolyte interface.

To gain insight on the doping state of PEDOT in PEDOT:PSS:PLL OECTs we measured the transmitted intensity of each sample by varying V_G. The doping level of PEDOT determines its optical absorption and hence the k coefficient of the Lambert law in Supplementary Equation (23). Since the absorption at 620 nm increases as the PEDOT is de-doped[5] we tracked the doping level of the semiconductor by calculating k when V_G ranges from 0 V to 0.5 V (V_S = V_D = 0 V) according to the following equation:

$$\frac{k_{\mathrm{V_G}}}{k_{\mathrm{V_0}}} = \frac{\log\left(\frac{I_{\mathrm{V_0}}}{I_{\mathrm{V_G}}}\right)}{t_{\mathrm{x}}k_{\mathrm{V_0}}c_{\mathrm{x}}} + 1 \tag{25}$$

where I_{VG} and I_{V0} are the measured transmitted intensities at a given V_G and V_G = 0, respectively and k_{VG} and k_{V0} are the molar attenuation coefficient at 620 nm at a given V_G and V_G = 0 V, respectively. Supplementary Figure 9 shows k_{VG}/k_{V0} as a function of V_G for OECTs with several PLL contents. The characteristic is fairly the same for all the measured devices, except for the device with PEDOT:PSS/PLL equal to 1/50, which shows no absorption variation due to the negligible content of PEDOT in the deposited film. This confirms that the

doping level of the PEDOT in the films is independent of the PLL content, providing a more solid evidence that small amounts of PLL added to the dispersion results in an electrostatic compensation in the bulk of the semiconductor without affecting the polyelectrolyte/semiconductor interface. Moreover, when the PLL content is high enough to compensate the fixed charge at the interface, the involved semiconductor separates from the dispersion, while the PEDOT remaining in the dispersion shows no variation in its doping level.

SUPPLEMENTARY NOTE 4: Inverter simulation framework

The device model implemented in the circuit simulator is derived from the Friedlein drain current model including the potential at the polymer/electrolyte interface contribution and a λ parameter accounting for the channel length modulation[10]. The model equations are:

$$I_{\mathrm{D}} = \frac{\Gamma}{\gamma} V_{\mathrm{P}}{}^{2-\gamma}[(V_{\mathrm{T}} - V_{\mathrm{G}} + V_{\mathrm{D}})^{\gamma} - (V_{\mathrm{T}} - V_{\mathrm{G}} + V_{\mathrm{S}})^{\gamma}][1 + \lambda(V_{\mathrm{S}} - V_{\mathrm{D}})] \quad \text{when } V_{\mathrm{T}} - V_{\mathrm{G}} \geq -V_{\mathrm{D}} \tag{26}$$

$$I_{\mathrm{D}} = -\frac{\Gamma}{\gamma} V_{\mathrm{P}}{}^{2-\gamma}(V_{\mathrm{T}} - V_{\mathrm{G}} + V_{\mathrm{S}})^{\gamma}[1 + \lambda(V_{\mathrm{S}} - V_{\mathrm{D}})] \quad \text{when } V_{\mathrm{T}} - V_{\mathrm{G}} < -V_{\mathrm{D}} \tag{27}$$

where

$$\Gamma = \frac{Wt}{L}\mu C_{\mathrm{v}} \tag{28}$$

$$\gamma = \frac{E_0}{k_{\mathrm{B}}T} + 1 \tag{29}$$

$$V_{\mathrm{T}} = V_{\mathrm{P}} - V_{\mathrm{SH}} \tag{30}$$

$$V_{\mathrm{SH}} = V_{\mathrm{G/E}} + V_{\mathrm{E/P}} \tag{31}$$

$$V_{\mathrm{G/E}} = \frac{k_{\mathrm{B}}T}{q} \log c \tag{32}$$

$$V_{\mathrm{E/P}} = -\frac{k_{\mathrm{B}}T}{q} \operatorname{asinh}\left(\frac{z_{\mathrm{fix}} N_{\mathrm{fix}}}{2zc}\right) \tag{33}$$

The value for the λ parameter is extracted by fitting the measured output characteristics of an OECT at several V_{G} and electrolyte concentrations as shown in Supplementary Figure 10, yielding $\lambda = 0.07\ \mathrm{V}^{-1}$.

The OECT channel is Crys-P and tin alloy (STANNOL Flowtin TSC263) is used as a non-polarizable gate electrode for easy integration and control of the electrolyte potential in OECTs. The measured potential at the tin/electrolyte interface is 496 ± 4 mV with respect to the Ag/AgCl pellet.

Supplementary references

[1] Bernards, D. A. & Malliaras, G. G. Steady-state and transient behavior of organic electrochemical transistors. *Adv. Funct. Mater.* **17**, 3538–3544 (2007).

[2] Bernards, D. A. *et al.* Enzymatic sensing with organic electrochemical transistors. *J. Mater. Chem.* **18**, 116–120 (2008).

[3] Giovannitti, A. *et al*. Controlling the mode of operation of organic transistors through side-chain engineering. *Proc. Natl Acad. Sci. USA* **113**, 12017–12022 (2016).

[4] Nielsen, C. B. et al. Molecular design of semiconducting polymers for high-performance organic electrochemical transistors. *J. Am. Chem. Soc*. **138**, 10252–10259 (2016).

[5] Friedlein, J. T., Shaheen, S. E., Malliaras, G. G. & McLeod, R. R. Optical measurements revealing nonuniform hole mobility in organic electrochemical transistors*. Adv. Electron. Mater*. **1**, 1500189 (2015).

[6] Rivnay, J. *et al.* High-performance transistors for bioelectronics through tuning of channel thickness. *Sci. Adv*. **1**, e1400251 (2015).

[7] Cetin, M. Electrolytic conductivity, Debye-Hückel theory, and the Onsager limiting law. *Phys. Rev*. *E* **55**, 2814-2817 (1997).

[8] Lin, P., Yan, F. & Chan, H. L. W. Ion-sensitive properties of organic electrochemical transistors. *ACS Appl. Mater. Interfaces* **2**, 1637–1641 (2010).

[9] Na, S.-I. *et al.* Evolution of nanomorphology and anisotropic conductivity in solvent-modified PEDOT:PSS films for polymeric anodes of polymer solar cells. *J. Mater. Chem*. **19**, 9045–9053 (2009).

[10] Torricelli, F. *et al*. Transport Physics and Device Modeling of Zinc Oxide Thin-Film Transistors - Part II: Contact Resistance in Short Channel Devices. *IEEE Trans. Electron. Dev*. **58**, 3025-3033 (2011).
